# Group 2 Innate Lymphoid Cells and the House Dust Mite-Induced Asthma Mouse Model

**DOI:** 10.3390/cells9051178

**Published:** 2020-05-09

**Authors:** Yuichiro Yasuda, Tatsuya Nagano, Kazuyuki Kobayashi, Yoshihiro Nishimura

**Affiliations:** Division of Respiratory Medicine, Department of Internal Medicine, Kobe University Graduate School of Medicine, 7-5-2 Kusunoki-cho, Kobe, Hyogo 650-0017, Japan; yyasuda@med.kobe-u.ac.jp (Y.Y.); kkoba@med.kobe-u.ac.jp (K.K.); nishiy@med.kobe-u.ac.jp (Y.N.)

**Keywords:** asthma, house dust mite, innate lymphoid cell 2, mouse model

## Abstract

Asthma is an important issue not only in health but also in economics worldwide. Therefore, asthma animal models have been frequently used to understand the pathogenesis of asthma. Recently, in addition to acquired immunity, innate immunity has also been thought to be involved in asthma. Among innate immune cells, group 2 innate lymphoid cells (ILC2s) have been considered to be crucial for eosinophilic airway inflammation by releasing T helper 2 cytokines. Moreover, house dust mites (HDMs) belonging to group 1 act on airway epithelial cells not only as allergens but also as cysteine proteases. The production of interleukin-25 (IL-25), IL-33, and thymic stromal lymphopoietin (TSLP) from airway epithelial cells was induced by the protease activity of HDMs. These cytokines activate ILC2s, and activated ILC2s produce IL-5, IL-9, IL-13, and amphiregulin. Hence, the HDM-induced asthma mouse model greatly contributes to understanding asthma pathogenesis. In this review, we highlight the relationship between ILC2s and the HDM in the asthma mouse model to help researchers and clinicians not only choose a proper asthma mouse model but also to understand the molecular mechanisms underlying HDM-induced asthma.

## 1. Introduction

Asthma is a heterogeneous disease, usually characterized by chronic airway inflammation. It is defined by the history of respiratory symptoms such as wheeze, shortness of breath, chest tightness, and cough that vary over time and in intensity, together with variable expiratory airflow limitation [1]. A diagnosis of asthma requires the presence of reversible airflow obstruction [2]. Airway inflammation involves inflammatory cells, such as eosinophils, neutrophils, lymphocytes, and mast cells; and airway components, such as bronchial epithelial cells, fibroblasts, and bronchial smooth muscle cells, and various humoral factors. Persistent airway inflammation causes remodeling and results in irreversible airflow limitations [3]. Worldwide, up to 300 million people suffer from asthma, including children. In particular, asthma is the most common chronic disease of childhood [1]. Although asthma-related deaths are declining in the United States [4], the total annual cost of asthma is increasing and reached 81.9 billion USD in 2013 [5]. Yaghoubi et al. also showed that the direct costs of asthma in adolescents and adults in the United States during the next 20 years was estimated to be 1,537 billion USD [6]. The use of biological agents, such as omalizumab, mepolizumab, benralizumab, and dupilumab, has been approved for severe asthma, which may lead to an increased cost associated with asthma [7,8,9,10]. In addition, asthma patients visit emergency departments and sometimes need hospitalization, which forces patients to leave school or work. Hence, the economic burden of asthma is significantly high [11]. As some specific biologics are used for severe asthma, the pathogenesis of asthma is gradually being elucidated. However, not everything about asthma pathogenesis has been elucidated. Moreover, severe asthma patients have become a major clinical problem. In a Dutch study, 3.6% of asthma patients over 18 years met the definition of severe asthma [12]. Severe asthma is not only costly and expensive but also increases the burden on the family [13]. Therefore, further elucidation of the pathogenesis of asthma and the establishment of treatment for severe asthma are urgent issues. From previous immunological studies of asthma, the endotypes of asthma are classified as type 2 immune responses and non-type 2 immune responses [14,15]. The type 2 immune response is composed of T helper 2 (Th2) cells and group 2 innate lymphoid cells (ILC2s).

In this paper, first, we give an overview of the classification of ILCs, and then explain the general mechanism of asthma, and describe the detail of the involvement of ILC2 and asthma. Finally, we shed light on the house dust mite (HDM)-induced asthma mouse model, which is a physiological asthma mouse model that is more suitable for analyzing the innate type 2 immune response involving ILC2s. This review will help researchers and clinicians to not only choose a proper asthma mouse model but also to understand the molecular mechanism underlying HDM-induced asthma.

## 2. ILC Group Classification

ILCs are a new group of lymphocyte immune cells. Unlike T cells and B cells, they do not have an antigen receptor. Therefore, they cannot cause an antigen-specific immune response. On the other hand, they have receptors for various cytokines, lipid mediators, and neuropeptides, and can produce large amounts of cytokines in several hours in response to various signals produced by surrounding cells. ILCs are mainly classified into three subtypes according to transcription factors and produced cytokines. ILC1 expresses T-bet and contributes to immune response to the virus by producing interferon-γ. ILC2 expresses GATA3, and produces IL-5 or IL-13. ILC2 is involved in parasite elimination and the development and exacerbation of allergic diseases. ILC3 expresses RORγ and produces IL-17 and IL-22. ILC3 is involved in bacterial elimination and the development and exacerbation of autoimmune diseases [16,17]. Therefore, it is expected that in the future, the etiology of various diseases will be elucidated and treatment targeting ILCs will be established.

## 3. Immunopathogenesis of Asthma

**Figure 1:**
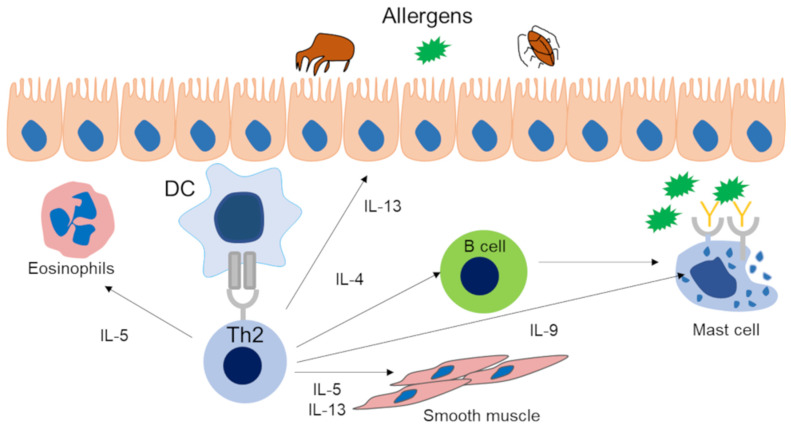
Type 2 immune response via Th2 lymphocytes in asthma patients. Dendritic cells capture inhaled antigens and present them to CD4-positive T cells via the T cell receptor. As a result, cytokines such as IL-4, IL-5, IL-9, and IL-13 are released from Th2 cells. IL-4 is involved in the differentiation of B cells into IgE-producing cells. IL-5 causes eosinophil activation and tissue eosinophilia. IL-9 is involved in mast cell proliferation. IL-13 causes goblet cell metaplasia. IL-5 and IL-13 cause bronchial hyperreactivity. DC, dendric cell; Th2, T helper 2 cell.

**Figure 2:**
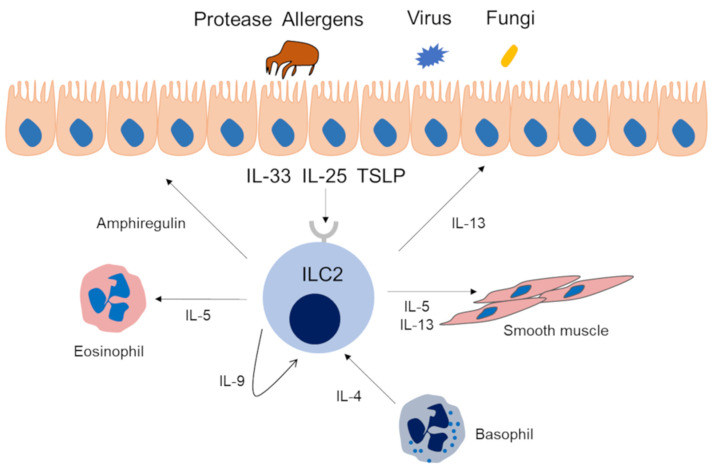
Type 2 immune response via ILC2s in asthma patients. Allergens with protease activity, fungi, and viruses promote the production of IL-25, IL-33, and thymic stromal lymphopoietin (TSLP) by airway epithelial cells. These cytokines activate ILC2s, and activated ILC2s produce IL-5, IL-9, IL-13, and amphiregulin. IL-5 and IL-13 have the same effect as that of the Th2 cell pathway. IL-9 prolongs ILC2 survival by autocrine actions. Amphiregulin is involved in tissue repair.

For Th2 cells, dendritic cells (DCs) capture inhaled allergens and present them to cluster of differentiation (CD) 4-positive T cells. Subsequently, Th2 cells produce cytokines related to asthma, such as IL-5, IL-4 and IL-13. Eosinophils, basophils, and mast cells are activated by the cytokines secreted by these cells and are also involved in the type 2 immune response (Figure 1. It is noteworthy that basophils are not only effector cells; actually, they may contribute to prime the Th2 adaptive response through early and significant production of IL-4, which is important to drive the T cells towards a type 2 phenotype in respiratory allergy/asthma [18]. The cytokines IL-4, IL-5, IL-9, and IL13 are related to type 2 immunity. IL-4 is essential for the differentiation and proliferation of Th2 cells and the differentiation of allergen-specific B cells into IgE-producing cells and plays an important role in the development of immediate allergic reactions [19,20]. In addition, IL-4 induces mucin gene expression and goblet cell metaplasia. IL-5 is essential for the maturation of eosinophils in the bone marrow and their release into the blood. IL-5 plays a role in the maturation, growth, activation, and survival of eosinophils. IL-9 is involved in mast cell proliferation and plays a crucial role in ILC2 survival [2,3]. IL-13 has various actions in asthma, including the expression of adhesion molecules such as vascular cell adhesion molecule 1 (VCAM-1) in vascular endothelial cells, goblet cell hyperplasia, increased mucus production, production of inducible nitric oxide synthase by airway epithelial cells, proliferation of airway smooth muscle, and stimulation of airway hyperresponsiveness [3]. On the other hand, IL-25, IL-33, and thymic stromal lymphopoietin (TSLP), which are mainly secreted from the airway epithelium due to *Alternaria*, protease activity of antigens such as HDM, and fungus and viral infection, are also involved in type 2 immunity. These cytokines induce IL-5 and IL-13 by activating ILC2s and cause airway inflammation [21] (Figure 2). The innate immune response is involved in the secretion of these cytokines from airway epithelial cells. Pathogen-associated molecular patterns, and damage-associated molecular patterns and their receptors, pattern recognition receptors (PRRs), play important roles in innate immune responses. PRRs are present in airway epithelial cells, macrophages, and dendritic cells in the respiratory tract. Among them, toll like receptor (TLR), which is one of the PRRs, has been suggested to be associated with asthma [22]. Group 2 HDM and *Aspergillus*-derived fibrinogen cleavage products induce allergic airway inflammation via TLR4 of airway epithelial cells [23]. Moreover, detailed analysis of the relationship between HDM and the innate immune response have been reported [24,25,26]. Since the related PRRs vary depending on the allergen, it is also necessary to pay attention to the involvement of the innate immune response in understanding the pathology of asthma.

Intriguingly, some asthma patients show a neutrophil-predominant phenotype irrespective of Th2 cytokines. The non-type 2 immune response seems to involve IL-17, which is associated with blood and sputum neutrophils and asthma severity [15]. Moreover, in a mouse model, Th17-induced inflammation and airway hyperresponsiveness showed steroid resistance [27]. Hence, Th17-mediated airway inflammation contributes to severe and difficult-to-treat asthma.

Asthma is thought to develop due to not only environmental factors but also genetic factors. In recent years, a genome-wide association study (GWAS) was conducted to search for genetic factors associated with asthma, and various candidate genes have been identified [28]. Among them, IL-33 and its receptor ST2 were identified with asthma, which suggests the importance of the IL-33 pathway. In addition, TSLP was found to be significantly associated with Japanese adult asthma patients [29]. Therefore, GWAS analyses also showed the importance of the ILC2 activation pathway, which is mediated by cytokines derived from airway epithelial cells.

## 4. ILC2s and Asthma

In 2010 it was first reported that ILC2s were activated by epithelial cytokines such as IL-25 and IL-33 produced large amounts of IL-5 and IL-13 [30]. This first report also revealed a type 2 immune response independent of Th2 cells. ILC2s express GATA3, a master transcription factor for Th2 cells, and produce a large amount of Th2 cytokines, such as IL-4, IL-13, and IL-9, by reacting with IL-25, IL-33, and TSLP. ILC2s are present in most organs of the body but are considered to be particularly abundant in skin, lung, and adipose tissue [31]. Because ILC2s are involved in type 2 immunity, the impact on the onset of allergic immune reactions is highlighted. ILC2s can rapidly produce Th2 cytokines [32]. This allows ILC2s to initiate and amplify the immune response and affect both innate and adaptive immunity through secreted cytokines. Hence, it is suggested that ILC2s have a crucial role in asthma pathogenesis.

In fact, there have been some reports that human ILC2s are increased in the peripheral blood of patients with asthma; however, there are reports that showed opposite results [33,34,35]. This discrepancy might be due to the different methods to identify ILC2s and the different asthma populations. In general, ILC2s in peripheral blood tend to increase in severe refractory asthma and eosinophilic asthma. Although it has been reported that ILCs in bronchoalveolar lavage fluid (BALF) and sputum are increased in asthma patients, it is not clear how ILC2s in peripheral blood or airway are involved in the pathology of asthma [36,37]. Many studies have tried to elucidate the background mechanism between asthma and ILC2s.

Studies on the genetic factors in allergic diseases using GWAS have reported that IL-33, one of the factors that activates ILC2s, was the factor that was most highly correlated with the development of bronchial asthma [38]. IL-33 is present in the nucleus of epithelial cells and is released from epithelial cells in response to fungi such as *Alternaria*, protease activity of HDMs, or viral infection. Then, eosinophilic airway inflammation is induced by the type 2 cytokines produced by ILC2s [39]. Kondo et al. demonstrated that the administration of IL-33 into mice-induced airway hyperresponsiveness and goblet cell hyperplasia accompanied by increased eosinophil counts and type 2 cytokines independent of acquired immunity [40]. Hence, the IL-33/ILC2 axis could be involved in the induction of eosinophilic airway inflammation independently of IgE antibodies and Th cells. Furthermore, IL-33 is more potent than IL-25 in activating lung ILC2s and airway contraction [41]. TSLP has been shown to induce steroid resistance in lung ILC2s in mouse models. TSLP binds to the TSLP receptor on ILC2s and enhances the expression of the antiapoptotic molecule Bcl-xL via activation of STAT5. Of note, it has been reported that TSLP-STAT5-Bcl-xL pathway-induced ILC2s sustain high cytokine production upon IL-33 stimulation even in the presence of steroids [42]. In addition, the expression of TSLP and IL-33 is enhanced in the peripheral blood and lung tissue of patients with severe asthma [43,44]. TSLP has also been identified as an important disease gene of asthma in GWAS [45]. In clinical settings, tezepelumab, a human monoclonal antibody specific for TSLP, reduced the rate of asthma exacerbation in patients with uncontrolled asthma [46]. Recently, treatments targeting GATA3, a transcription factor in ILC2s and Th2 cells, have also been performed. SB010, a DNA enzyme that is capable of cleaving and inactivating GATA3 messenger RNA, significantly attenuated the early and late phases of asthmatic responses after allergen induction in patients with allergic asthma. On the other hand, biomarker analysis showed that plasma levels of IL-5 were attenuated [47]. Thus, in recent years, the pathogenesis of bronchial asthma has been elucidated not only in the acquired immune system in association with Th2 cells but also in the innate immune system, such as ILC2s and cytokines, from epithelial cells.

## 5. Ovalbumin-Induced Asthma Mouse Model

Since OVA is inexpensive and available in large quantities, it has frequently been used as a protein antigen to induce allergies in animal models. The OVA-induced asthma mouse model generally induces airway inflammation by administration of OVA to mice to establish sensitization and then challenge by inhalation of OVA into the airways. This mouse model is suitable for analysis of the type-2 immune response. In general, sensitization is performed by intraperitoneal and subcutaneous routes. However, it is difficult to promote the development of the Th2 phenotype by the immune response to a single administration of OVA [48]. Therefore, adjuvants such as aluminum hydroxide (AlOH_3_) are required to induce airway inflammation [49]. The OVA-induced asthma mouse model is a traditional method, and there are many reports to date. However, OVA has been replaced by aeroallergens, such as HDM, in recent years. This is because intraperitoneal and subcutaneous routes to sensitize animals are not similar to human sensitization. Moreover, intraperitoneal routes might induce immune tolerance. Therefore, there is a trend to use intranasal administration of antigens, which has the advantage of not requiring adjuvants and is similar to human sensitization [50].

## 6. *Aspergillus fumigatus* Allergen-Induced Asthma Mouse Model

In addition to the OVA-induced asthma mouse model, there are also *Aspergillus fumigatus* (Af) allergen-induced asthma mouse models. Af extracts contain not only protein antigen but also proteases or ligands for innate immune cells [51]. This model is not clearly divided between sensitization and exposure period like the OVA model, but is established by continuous intranasal administration twice a week for 8 weeks or three times a week for 3 weeks [52,53]. This mouse model is also suitable for analysis of the type-2 immune response.

## 7. House Dust Mites and Asthma

Although only 4 from more than 30 of HDM allergens are proteases, HDMs are characterized by protease activity, immunogenicity, and induction of the innate immune system [24]. In general, actions to prevent asthma can be divided into three stages. Primary prevention involves preventing the development of asthma and should be performed in children before allergen sensitization. Secondary prevention involves preventing the onset of asthma after sensitization mainly by allergen exposure. Tertiary prevention involves preventing exacerbation after the onset of asthma, which prevents respiratory function decline and asthma death. Allergens, including HDMs, are considered risk factors at all stages of prevention. Sensitization to indoor allergens is more important than outdoor allergens for the development of asthma [1]. Among children, exposure to HDMs are associated with increased risks of asthma [54]. Moreover, HDMs are a risk factor for asthma exacerbation [55]. Thus, there is a close relationship between asthma and HDMs.

HDMs are a major risk factor for allergic diseases, such as atopic dermatitis (AD), allergic rhinitis (AR), and asthma [56]. They are found in dust, mattresses, pillows, and bedding [57]. The life cycle of HDMs from egg to adult takes 3 to 4 weeks, and they live for approximately 2 months. Females lay approximately 80 eggs during this time. Mite allergens have been classified into more than 30 groups [24]. In particular, *Dermatophagoides pteronyssinus* (Der p) and *Dermatophagoides farinae* (Der f) are the most common sources of indoor allergens. The prevalence of HDM allergen sensitization varies from 65 to 130 million persons in the general population [20]. Although there are geographical differences, more than 80% of pediatric asthma patients in Taiwan had HDM sensitization [58]. The allergenic potential of HDMs is due to their dead bodies and their fecal pellets, which have protease activity. Moreover, components of HDMs include lipopolysaccharide (LPS), β-glucan, and chitin [59]. Based on the above, it seems that various components of HDMs activate the immune system.

With recent technological advances, the allergenic effects of HDMs have been identified. Two allergen biological activities, which are proteolysis and peptidelipid/lipid binding, induce IgE and stimulate bystander responses to unrelated allergens [24].

## 8. ILCs and HDMs

As mentioned above, the components of HDMs vary. Therefore, various innate immune responses are involved in a complex manner.

It is noteworthy that Der p 1 and Der f 1 are cysteine proteases [25,26]. Der p 1 cleaves tight junctions by proteolysis of the extracellular domains of occludin and non-pore-forming claudins. The tight junctions repair quickly because synthesis of occludin occurs quickly after the initiation of tight junction cleavage. The reversible cleavage of epithelial tight junctions results in increased epithelial permeability [24,26]. Cysteine proteases destroy the tight junctions between airway epithelial cells, which is thought to contribute to the development of allergic disorders [60,61,62]. Cysteine proteases cause allergic symptoms by acting on ILC2s in an independent manner [63] and are known to strongly induce the production of IL-33 by inducing necrosis of epithelial cells [64]. Furthermore, IL-13, derived from ILC2s, is also involved in antigen presentation of Th2 cells by activating macrophages and dendritic cells. Actually, it has been shown that the pathology of chronic inflammation by Th2 cells is suppressed in mice that lack ILC2s. Therefore, this result suggests that ILC2s are important not only for the initial response of inflammation but also for chronic inflammation [65]. IL-33 activates ILC2s, which induce the production of IL-5, IL-4, IL-9, IL-13, and amphiregulin [31]. As a result, type 2 airway inflammation occurs. As for TLR4 and HDM, thrombin is produced by the prothrombinase activity of the group 1 HDM allergen. As a result, activation of protease-activated receptor (PAR)-1 and PAR-4 initiates reactive oxidant species (ROS) production, and finally IL-33 is produced [25]. In addition, chitin induces the expression of IL-25, IL-33, and TSLP in airway epithelial cells, then activates ILC2 by these cytokine released IL-5 and IL-13 [66]. Moreover, Choi et al. revealed that airway hypersensitivity to HDR-derived chitin is mediated by the TLR2-dependent pathway [67].

In clinical studies of allergic rhinitis (AR), Zhong et al. demonstrated that ILC2 levels were increased in the peripheral blood of patients with AR who were sensitized to HDM. In addition, ILC2s in AR patients produce more IL-5 and IL-13 after stimulation with epithelial cytokines. These data indicate that ILC2s have a critical role in antigen-positive AR patients and that ILC2s might be involved in HDM-AR [68].

## 9. The HDM-Induced Asthma Mouse Model

### 9.1. Overview of the Model

Animal models of asthma are useful tools for understanding the pathogenesis of asthma. To date, many mouse models of allergic airway inflammation have been established to elucidate the different characteristics of asthma. Each asthma mouse model differs in the type of allergen used, the strain of the mouse, the administration period, and the dose of the allergen. As common allergens, OVA, HDM, cockroaches, and *Alternaria* are often used [69]. Recently, asthma mouse models induced by proteases, such as papain or cytokines, including IL-25- and IL-33, have also been established [21,41,70]. The selection of the mouse model varies depending on the clinical parameter to be measured. To date, asthma mouse models using OVA have been widely used for the analysis of asthma pathology. However, a mouse model that induces asthma by administering HDM extract, which is the main cause of human asthma, has recently been used for the analysis of asthma pathology because of the similarity of symptoms with human asthma. Even in the same HDM-induced asthma mouse model, it is possible to create an acute asthma model, a chronic asthma model, and a remodeling model depending on the amount of antigen exposure and the administration period [71,72,73]. By using these models, it is possible to express eosinophilic airway inflammation, airway hyperresponsiveness and tissue remodeling, which are characteristic of asthma. Therefore, HDM-induced asthma mouse models have the advantage of being able to establish various phenotypes. Recently, the association between ILC2s and HDM-induced asthma mouse models has been analyzed. In this section, we review what has been found so far in the HDM-induced asthma mouse model. In addition, we provide a summary of experimental protocols of HDM induced murine asthma models (Table 1).

However, this model has an important limitation of variations in nature of the HDM extract itself. Post et al. evaluated the biochemical properties of three commercially available HDM extracts by in vitro and in vivo methods. They demonstrated that the different HDM extracts varied in their biochemical activities and induced different responses in vitro and in vivo [74]. Therefore, it is necessary to judge each study based on the above. Therefore, each study must be interpreted based on the differences in the nature of HDM.

### 9.2. Th2 Cytokine Production Associated with ILC2 Activation

In 2012, Klein et al. published a paper on pulmonary ILCs and Th2 cytokines. BALB/c mice were intranasally sensitized with 100 μg HDM and intranasally challenged either once, three, or ten times with 10 μg HDM. HDM-challenged mice showed increased cell numbers, eosinophilia, and T cell influx in bronchoalveolar lavage fluid (BALF). Moreover, these mice showed an increase in ILC2s in the lungs and BALF. Interestingly, intracellular flow cytometry demonstrated that ILC2s in the lung in the HDM group produced IL-5, IL-13, and IL-4. However, IL-4 production in ILC2s was limited and slight compared to the increase in Th2 cells. On the other hand, IL-5 and IL-13 produced by ILC2s were almost comparable to those produced by Th2 cells. Therefore, the researchers concluded that ILC2s contributed to allergic airway inflammation in asthma by producing IL-5 and IL-13 to the same extent as Th2 cells [75]. However, in 2012, the opposite result was reported by Taylor et al. Six- to 8-wk-old male and female C57BL/6 mice were intranasally challenged once with 100 μg of *Alternaria*, HDM, *Aspergillus fumigatus* or *Candida albicans*. Except for the *Alternaria* group, all mice were analyzed after 12 h. Only the *Alternaria* group was analyzed after 3 h, 6 h, 12 h, and 3 days. An increase in eosinophils, IL-33, IL-5, and IL-13 in BALF was observed only in *Alternaria*-challenged mice [76]. There seem to be several factors underlying this opposite result. One of the factors was the difference in the number of allergens administered between the two studies. In addition, there may be differences in the timing of the analysis. Furthermore, in a study using human airway epithelial cells (BEAS-2B cells), IL-13 expression peaked approximately 2 h after stimulation with HDM [78]. These factors may explain this discrepancy.

### 9.3. ILC2 Responses to Local Allergens

In 2014, Matthew et al. demonstrated that ILC2s played an important role in the development of an acquired type 2 response to local but not systemic antigen exposure. Wild-type (WT) and ILC2-deficient C57BL/6J mice were sensitized on days 0, 1, and 2 with 25 μg of HDM and challenged on days 14, 15, 16, and 17 with 5 μg of HDM. All mice were sacrificed 24 h after the final challenge. In this study, a well-established OVA-induced asthma mouse model was used as a systemic antigen model. For the HDM-induced asthma mouse model, ILC2-deficient mice showed a decrease in eosinophils in BALF and a decrease in lung ILC2s; both total serum IgE and HDM antigen-specific IgE and IgG1 levels were significantly reduced, and the expression of lung transcripts of IL-4 and IL-5 was significantly reduced compared to that of WT mice. In contrast, in the OVA-induced mouse model, there was no difference in serum IgE or IL-5 expression or the number of eosinophils in BALF. Thus, ILC2s have a crucial role in the initiation of the Th2 immune response to the mucosal route of antigen exposure [77].

### 9.4. The Difference between Murine and Human ILC2

There are limitations regarding differences between murine and humans ILC2. Mouse and human ILC2s are phenotypically similar, with some common surface markers being expressed [79]. However, mouse ILC2s also express KLRG1 and ICOS, whereas human ILC2s express CRTH2 and CysLT1R [80]. Therefore, it should be noted that different surface markers may cause the different ILC2 activation pathways.

## 10. The Difference of ILC2 Response in HDM, OVA and *Aspergillus fumigatus*-Induced Asthma Mouse Models

As mentioned in Section 8, recent studies have revealed that the mechanism of ILC2 activation by HDM involves the innate immune response. Therefore, we also considered the relationship between ILC2 and innate immune response in other mouse models. There was a report that ILC2s were a major production source of Il-5 and IL-13 in an OVA-induced asthma mouse model [75]. However, it should be noted that this study differed from the conventional OVA mouse model in that mice were challenged with OVA after intravenous injection of Th2-polarized T cells. Other reports showed that ILC2 was hardly induced in the OVA asthma mouse model [42], and eosinophilic airway inflammation and IL-5 production were unchanged in ILC2-deficient mice [77]. These data suggest that the role of ILC2 as a source of Th2 cytokine production in a model of adaptive immunity may be limited. However, a recent study demonstrated that TLR2 signaling mediates a Th2-driven immune response to OVA [81]. Therefore, it is suggested that the innate immune response may be involved in the OVA-induced asthma mouse model, and further investigation is necessary to reveal these mechanisms. Regarding *Aspergillus*, it has been reported that intranasal administration of *Aspergillus oryzae* to mice showed ILC2 activation and eosinophilic airway inflammation as the same as papain [82]. However, there is no report on the involvement of ILC2 in the *Aspergillus fumigatus*-induced mouse model. Furthermore, stimulation of *Alternaria* or HDM in human nasal epithelial cells induced IL-33 and TSLP, but stimulation of *Aspergillus fumigatus* did not. However, Khosravi et al. showed that *Aspergillus fumigatus* had a potential ability not only to stimulate murine lung epithelial cells to produce IL-25, IL-33, and TSLP, but also to express TLR2 and TLR4 genes [83]. Therefore, *Aspergillus fumigatus* might activate ILC2 by an epithelial-derived cytokine via the TLR pathway. A summary of ILC2 responses of each murine asthma model is shown in Table 2.

## 11. Conclusions

We summarized previous findings on HDM and ILC2 and reviewed mouse models to evaluate them. Of course, this experimental model has various limitations including the difference between murine and human ILC2s, and the nature of the HDM extract itself. Moreover, it has been reported that IL-33 and HDM induce different ILC2 surface markers [84]. Therefore, it might be suggested that there are differences in the role of ILC2 in asthma mouse models due to allergen and cytokine exposure. However, the HDM-induced asthma mouse model is considered to be close to the human asthma model in pathophysiology, including its relationship to asthma, antigenicity, and action as a protease. Moreover, the allergic effect of HDM is being elucidated with recent technological advances. Hence, understanding of this model is important for elucidating asthma pathology. We hope this review will contribute to the further progress of asthma research in the future.

## Figures and Tables

**Table 1:** Experimental protocols of house dust mite-induced murine asthma models.

Reference	Mouse Strain	Protocol	Result
[75]	BALB/c	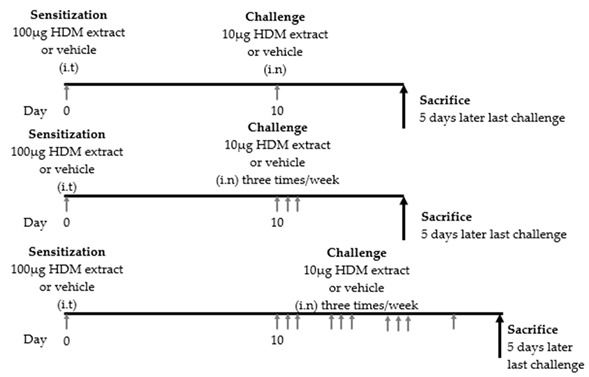	Increased ILC2 in lung and BALF Lung ILC2 produced IL-5, IL-13, IL-4 IL-5 + and IL-13 + cells accounted for a significant proportion of ILC2 in lung and BAL The contribution of ILC2 cells to the total population of IL-5 + and IL-13 + cells in the BAL was in the same range as seen for Th2 cells.
[76]	C57BL/6	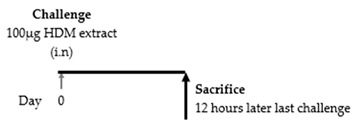	No elevation of IL-5, IL-13 and IL-33 in BAL
[77]	C57BL/6	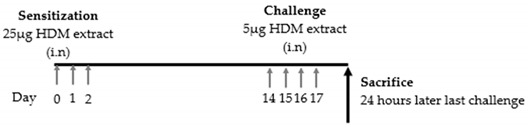	Elevation of eosinophils in BALF Elevation of lung ILC2, serum IgE and transcription level of IL-4 or IL-5 from lung RNA Leukocyte infiltration into the lung tissue, particular in the peribronchiolar and perivascular space.

i.t: intratracheal, i.n: intranasal, HDM; house dust mite, ILC2: group 2 innate lymphoid cell, IL: interleukin, BALF; bronchoalveolar lavage fluid, Th2:, T helper 2, RNA: ribonucleic acid.

**Table 2:** Summary of ILC2 response of each murine asthma model.

Reference	Model	Mouse Strain	Route	ILC2 Response
[75]	HDM	BALB/c	i.n	Increased ILC2 in lung and BALF
[77]	HDM	BALB/c	i.n	Elevation of lung ILC2
[75]	OVA	BALB/c	aerosol	Elevation of lung ILC2
[42]	OVA	C57BL/6	i.n	No elevation of lung ILC2
[77]	OVA	C57BL/7	i.n	No difference in eosinophilic inflammation and IL-5 production in ILC2-deficient mice
none	*A. fumigatus*	none	none	none

i.n: intranasal, *A. fumigatus*: *Aspergillus fumigatus*.
